# Changes in MRI head motion across development: typical development and ADHD

**DOI:** 10.1007/s11682-024-00910-w

**Published:** 2024-08-27

**Authors:** Phoebe Thomson, Vanessa Loosley, Emily Friedel, Timothy J. Silk

**Affiliations:** 1 Centre for Social and Early Emotional Development and School of Psychology, Deakin University, Geelong, VIC 3125 Australia; 2 Child Mind Institute, New York, NY 10022 USA; 3 Developmental Imaging, Murdoch Children’s Research Institute, Flemington Road, Parkville, VIC 3052 Australia

**Keywords:** Motion artifact, Longitudinal development, ADHD, Resting-state functional MRI, Diffusion MRI

## Abstract

Head motion is a major confounding variable for magnetic resonance imaging (MRI) analysis, and is commonly seen in individuals with neurodevelopmental disorders such as attention deficit hyperactivity disorder (ADHD). This study investigated the trajectory of change in head motion in typically developing children and children with ADHD, and examined possible altered trajectories in head motion between children with remitted and persistent ADHD. 105 children with ADHD and 84 controls completed diffusion and resting-state functional MRI scans at up to three waves over ages 9–14 years. In-scanner head motion was calculated using framewise displacement, and longitudinal trajectories analyzed using generalized additive mixed modelling. Results revealed a significant age effect on framewise displacement where head motion decreased as age increased during both diffusion (*p* < .001) and resting-state functional MRI (*p* < .001). A significant effect of group was also observed; children with ADHD displayed greater framewise displacement than controls over the age range (diffusion MRI *p* = .036, functional MRI *p* = .004). Further analyses revealed continued elevation in head motion in children in remission from ADHD (diffusion MRI *p* = .020, functional MRI *p* = .011) compared to controls. Rates of change in head motion did not significantly differ between diagnostic groups. Findings indicate a critical link between in-scanner head motion and developmental age within children regardless of ADHD diagnosis, important to consider in studies of neurodevelopment. Findings also suggest change in head motion with age does not differ between individuals with remitted and persistent ADHD, adding further evidence that behavioral manifestations of ADHD may continue despite clinical remission.

**Supplementary Information:**

The online version contains supplementary material available at 10.1007/s11682-024-00910-w.

Head motion is a major confounding variable in the analysis of magnetic resonance imaging (MRI) (Oldham et al., 2020; Murphy et al., 2013). Excessive motion produces artifacts in MRI images, leading to data loss, and costs to participant/researcher time and MRI resources. Currently, motion-related data loss can be reduced using a range of pre-and-post-collection techniques, including mock scanner training sessions, behavioral interventions, and use of natural sleep or sedation (Greene et al., 2018). Indices of in-scanner motion such as framewise displacement (FD; a measure of how much movement occurs from one frame to the next) may be used as exclusion criteria to remove or correct data distorted by excessive motion before MRI analysis (Baum et al., 2018; Power et al., 2012), though there is no gold standard on specific motion thresholds. Importantly, when conducting neuroimaging analyses across development or between clinical groups with different degrees of motion, significant problems can arise such as the lack of generalizability of findings due to the phenotype of excluded participants, and/or unequal distribution of data removal across study groups. Furthermore, even in usable MRI data small head movements can result in spurious biases in derived MRI measures (Power et al., 2012; Yendiki et al., 2014). For example, in examining functional connectivity across the brain, Power et al. (2012) found that higher head motion caused systematic but spurious effects, decreasing long-distance correlations and increasing short-distance connectivity correlations. The effects of motion on diffusion MRI (dMRI) have received far less attention than fMRI, despite awareness of the associated problems since early dMRI applications (Anderson & Gore, 1994). Motion introduces model-fitting biases in dMRI, and movement-related data contamination may persist even after employing motion-correction techniques (Baum et al., 2018; Ling et al., 2012; Yendiki et al., 2014).

Across development, it is notable that children have more extreme and persistent head movement during MRI scanning than adults (Pardoe et al., 2016). Independent studies using cross-sectional data have found that the intensity and frequency of motion during scanning is on average 0.50 mm during middle childhood (Power et al., 2012; Wilke, 2012), 0.09 mm during adolescence (Satterthwaite et al., 2012), and 0.05 mm in adulthood (Van Dijk et al., 2012). Problematically, Satterthwaite et al. (2012) investigated the association between head motion and age, and found use of multiple motion correction techniques (including mock scans and head stabilizations) does not eliminate or weaken this relationship. Moreover, when motion was not accounted for (particularly in neurodevelopmental research), results were biased and caused estimates of age effects on brain function to inflate due to motion and age producing similar effects (Satterthwaite et al., 2012). A recent study indicates that changes in motion across development are similarly problematic for dMRI, where age-related differences systematically bias estimates of white matter microstructure even after rigorous data quality assurance and retrospective head-motion correction (Baum et al., 2018). Age-related head motion may adversely affect inferences regarding functional and structural connectivity across development through both inflation and reduction of estimates.

In addition to greater head motion in children, motion is frequently greater in individuals with neurodevelopmental disorders such as attention deficit hyperactivity disorder (ADHD) (Pardoe et al., 2016). Current ADHD research highlights significant concerns regarding data loss or distortion caused by motion during MRI scanning, and ADHD symptoms have been directly linked to higher head motion during r-fMRI and dMRI sequences (Aoki et al., 2018; Couvy-Duchesne et al., 2016; Kong et al., 2014; Thomson et al., 2021). Despite this, systematic reviews and meta-analyses have revealed that a considerable proportion of dMRI studies on ADHD have failed to account for group differences in motion or even report correcting for head motion (Aoki et al., 2018; van Ewijk et al., 2012). Collectively, these studies provide initial evidence that head motion may be part of the ADHD phenotype, and further investigation is required to ensure correct interpretation of developmental ADHD research. Relatedly, reduction in ADHD symptoms and remission from an ADHD diagnosis are commonly seen as children develop into late adolescence and adulthood (Biederman et al., 2000; Willcutt, 2012). However, despite no longer meeting diagnostic criteria, individuals in remission from ADHD often still exhibit impaired functioning and symptoms associated with the disorder (Halperin et al., 2008; Thomson et al., 2020). It remains unknown how changes in ADHD diagnostic status relate to changes in head motion across development, and whether remission from ADHD symptoms is reflected in motion decreases towards typical levels.

While existing literature has indicated that during development head motion decreases with age, this study is the first to investigate this association longitudinally in children with and without ADHD in both r-fMRI and dMRI (Aim 1). It is hypothesized that as typically developing children age their framewise displacement will decrease. A similar decrease in framewise displacement is expected in children with ADHD, however children with ADHD are predicted to have consistently higher framewise displacement across the age range compared to children without ADHD. The current study further aimed to examine whether the trajectory of change in head motion during r-fMRI and dMRI scanning differs in children in remission from ADHD compared to children with persistent ADHD and controls (Aim 2). It is hypothesized that children in remission from ADHD will display an altered trajectory of head motion development, with greater decreases in framewise displacement towards the level of non-ADHD controls.

## Methods

### Participants

Data were collected as part of the Neuroimaging of the Children’s Attention Project, a community-based longitudinal study centered around symptom development and outcomes of children with and without ADHD, approved by the Royal Children’s Hospital Human Research Ethics Committee in Melbourne (see Sciberras et al., 2013; Silk et al., 2016 for full cohort details). Participants were originally recruited between ages 6–8 years from socio-economically diverse elementary schools across Melbourne, Australia. Participants with an intellectual disability (IQ < 70), serious medical condition (e.g., kidney disease), genetic disorder, moderate-severe sensory impairment, or neurological disorder were not included in data collection. Aligning with 36-month follow-up, participants were invited into a neuroimaging sub-study and followed for up to three waves at 18-month intervals (wave 1: 9.4–11.9 years, wave 2: 10.7–13.5 years, wave 3: 12.2–14.5 years). The Diagnostic Interview Schedule for Children (DISC-IV; Shaffer et al., 2000) was administered at recruitment, wave 1 and wave 3 to assess ADHD diagnostic status. The control group comprised individuals who did not meet criteria for ADHD at any timepoint. Participants who met criteria for ADHD at recruitment and/or wave 1 were part of the ADHD group. To examine effects of diagnostic remission, ADHD individuals were in the ADHD-remitted group if they met one of two conditions: (1) no longer met diagnostic criteria at wave 3; (2) no longer met diagnostic criteria at waves 1 and 3. Participants who did not have complete diagnostic information at wave 3 (for whom persistent or remitted status could not be determined) were excluded from analyses investigating the effect of diagnostic remission. Individuals continuing to meet ADHD diagnostic criteria were in the ADHD-persistent group (see supplementary materials page S2 for further information). To assess children in their usual classroom condition, participants currently using medication were asked to take medication as they would on a typical school day. ADHD medication use on assessment day was recorded, with 28 participants taking ADHD medication for at least one timepoint (see Table S1). Participants were included in the final sample if they had complete diagnostic (DISC-IV), MRI head motion and demographic information (i.e., age, sex, ADHD medication use) for at least one scan. All participating families provided written informed consent prior to data collection.

### Procedures

Children and their families attended an appointment at the Murdoch Children’s Research Institute, Melbourne, Australia. For familiarization with the MRI environment, participants completed a 30-minute training session in a mock MRI scanner. Following practice scanning, MRI was conducted using a 3-Tesla Siemens MRI scanner (Erlangen, Germany). Waves 1 and 2 were collected on a TIM Trio scanner; wave 3 was collected after an upgrade to a MAGNETOM Prisma scanner (scanner upgrade was accounted for within statistical modelling). For dMRI, High Angular Resolution Diffusion Imaging (HARDI) data was acquired with: TR/TE = 3200/110ms; voxel size = 2.4mm^3^; flip angle = 90°; FOV = 260 × 260 mm; 63 slices; multiband factor = 3; b = 2800 (60 directions, 4 volumes b = 0), 2000 (45 directions, 6 volumes b = 0), 1000 (25 directions, 6 volumes b = 0) sec/mm2; total acquisition time = 9 min 23s. Then, r-fMRI was acquired with: TR/TE = 1500/33ms, voxel size = 2.5mm^3^, flip angle = 85°, FOV = 255 × 255 mm, 60 slices, multiband factor = 3, acquisition time = 6 min 33s. During r-fMRI, participants were instructed to keep their eyes open and stare at a white fixation cross on a black screen. For calculating head motion parameters, mean framewise displacement (FD; Power et al., 2012) was calculated for each subject using the MCFLIRT function of the FMRIB Software Library (FSL, Version 6.0.0; Jenkinson et al., 2002). For r-fMRI, this calculation reflects the average amount of head motion present across three rotation and three translation parameters from one frame to the next; for dMRI the calculation reflects the mean absolute differences in consecutive rigid body (rotation and translation) parameters after aligning diffusion images to the reference b = 0 image. Images were visually inspected for extreme motion artifact (e.g., ghosting, venetian blind artifact), and scans excluded if present. As head motion was of primary interest, subjects were not excluded on the basis of head motion values.

### Statistical analysis

Head motion scores were modelled using generalized additive mixed models (GAMMs). GAMMs allow the best fitting model to take the form most appropriate for the data whether that be linear or non-linear, and are able to handle missing data and varying intervals between timepoints. Statistical testing was run using R (Version 4.2.1; R Core Team, 2022) statistical computing software, using the *gamm* function in the *mgcv* package (Wood, 2006). To assess effects of age and diagnostic status on motion, the following models were tested in order of increasing complexity:

1. FD∼1 + ADHDMedicationUse + Sex + ScanUpgrade.
2. FD∼*s*(Age, k = 4) + ADHDMedicationUse + Sex + ScanUpgrade.
3. FD∼*s*(Age, k = 4) + Group + ADHDMedicationUse + Sex + ScanUpgrade.
4. FD∼*s*(Age, k = 4) + Group + *s*(Age, k = 4,by = Group, m = 1) + ADHDMedicationUse + Sex + ScanUpgrade.

A log transformation was performed on mean FD due to a high degree of rightward skew. Age was grand mean centered to improve the interpretability of the model intercept. Group was defined as “ADHD” or “Control” for Aim 1, and “ADHD-Persistent”, “ADHD-Remitted” or “Control” for Aim 2. Sex, ADHD medication use, and scanner upgrade were included as covariates in all models. A random intercept and slope per participant were included in all models. A continuous autocorrelation structure with age was used, accounting for variability in time intervals between waves for each individual. Models 1–4 were compared using the Akaike information criterion, Bayesian information criterion and likelihood ratio test (Huang, 2017; Lewis et al., 2011). Models were initially estimated using maximum likelihood to allow for model comparison; restricted maximum likelihood was used to estimate final models to obtain accurate model parameters. For further information on model parameter specification see supplementary materials. See also supplementary materials for details of supplementary analysis splitting ADHD group by inattention or hyperactivity/impulsivity symptom criteria.

## Results

Demographic participant characteristics for the ADHD and Control groups are presented in Table 1. Age and socioeconomic status did not differ between groups, however the ADHD group had significantly more males (range *p* = .013–0.023) and lower IQ (*p* < .001). Participants attended on average 2.3 waves per person.

**Table 1:** Demographic characteristics by MRI modality

MRI	Measure	ADHD	Control	Test Statistic	*p*-value
Diffusion	Participants	103	83	-	-
	Males, *n* (%)	77 (75%)	49 (59%)	5.20	0.023
	Age, *M (SD)*				
	Wave 1	10.4 (0.5)	10.4 (0.4)	0.07	0.948
	Wave 2	11.7 (0.6)	11.7 (0.5)	0.34	0.738
	Wave 3	13.2 (0.6)	13.2 (0.5)	-0.54	0.594
	IQ, *M* (*SD*)	95 (13)	104 (14)	4.24	< 0.001
	SES, *M* (*SD*)	1019 (40)	1019 (47)	-0.05	0.960
	ADHD Medication use, *n* (%)	29 (28%)	-	-	-
Resting state functional	Participants	99	82	-	-
	Males, *n* (%)	75 (76%)	48 (59%)	6.11	0.013
	Age, *M (SD)*				
	Wave 1	10.4 (0.5)	10.4 (0.4)	-0.22	0.983
	Wave 2	11.7 (0.6)	11.7 (0.5)	0.33	0.739
	Wave 3	13.2 (0.6)	13.2 (0.5)	-0.54	0.594
	IQ, *M* (*SD*)	95 (14)	104 (14)	4.10	< 0.001
	SES, *M* (*SD*)	1018 (40)	1018 (47)	0.01	0.995
	ADHD Medication use, *n* (%)	28 (28%)	-	-	-

*Note* A total of 105 participants with ADHD and 84 Control participants had motion data for at least one scan modality

*M* = mean, *SD* = standard deviation, IQ = intelligence quotient, SES = socioeconomic status

### Effect of age and ADHD diagnosis on head motion

**Fig. 1:**
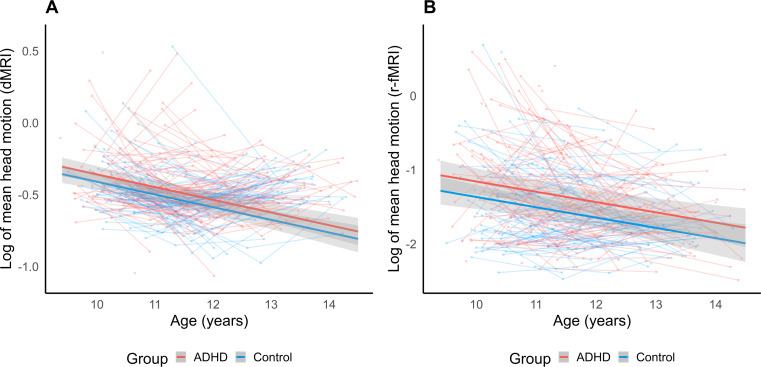
Longitudinal change in framewise displacement with age. *Note*. Figure depicts model trajectory of head motion (framewise displacement) change during (**A**) dMRI and (**B**) r-fMRI scans, plotted over raw motion values for each subject. Head motion decreases over age can be observed in both ADHD (red) and Control (blue) groups. Shaded areas represent 95% confidence intervals for best fitting model

Age and diagnostic status related change in head motion during dMRI and r-fMRI scans were assessed using GAMMs. After assessing the fit statistics for nested models, Model 3 was chosen as the best fitting model for both scan types (see Table S2). In this model, a significant effect of age was observed for FD scores across the whole sample whereby motion, separately during both dMRI and r-fMRI, decreased linearly as age increased (*p* < .001; see Table 2; Fig. 1). There was also a significant group effect, where the ADHD group displayed higher FD across the age range (r-fMRI *p* = .004; dMRI *p* = .036). There were no significant FD differences by sex or medication use, however a significant effect of scanner upgrade was observed for dMRI (*p* < .001). Models explain 9% of the variance in mean FD during both r-fMRI and dMRI. Supplementary analysis in individuals with ADHD revealed no significant effect of inattention or hyperactivity/impulsivity symptom levels on r-fMRI or dMRI FD (see Tables S3–4). Results are consistent after notch filtering framewise displacement values during r-fMRI as in Fair et al. (2020) (see Tables S6–7). Results are also consistent following exclusion of participants taking ADHD medication (see Tables S8–S10).

**Table 2:** Summary statistics of models demonstrating ADHD group effects on head motion over age

MRI	Parametric coefficients	Estimate	*SE*	*t*-value	*p*-value
Diffusion	(Intercept)	-0.50	0.03	-18.95	< 0.001
	Group (ADHD vs. Control)	-0.05	0.02	-2.10	0.036
	Scanner Upgrade	0.22	0.03	6.71	< 0.001
	Sex (Female vs. Male)	0.02	0.02	0.63	0.529
	ADHD Medication Use	0.04	0.04	1.06	0.288
	Smooth term	*edf*	*Ref.df*	*F*-value	*p*-value
	*s*(Age)	1	1	52.09	< 0.001
Resting state functional	Parametric coefficients	Estimate	*SE*	*t*-value	*p*-value
	(Intercept)	-1.38	0.08	-17.54	< 0.001
	Group (ADHD vs. Control)	-0.21	0.07	-2.91	0.004
	Scanner Upgrade	0.05	0.09	0.56	0.575
	Sex (Female vs. Male)	0.09	0.07	1.17	0.242
	ADHD Medication Use	-0.19	0.11	-1.81	0.071
	Smooth term	*edf*	*Ref.df*	*F*-value	*p*-value
	*s*(Age)	1	1	16.64	< 0.001

*Note* Adjusted *R*^*2*^ 0.09 for diffusion MRI and 0.09 for resting state fMRI

*SE* = Standard Error, *edf* = estimated degrees of freedom, *Ref.df* = reference degrees of freedom

### Effect of ADHD remission status on head motion

**Fig. 2:**
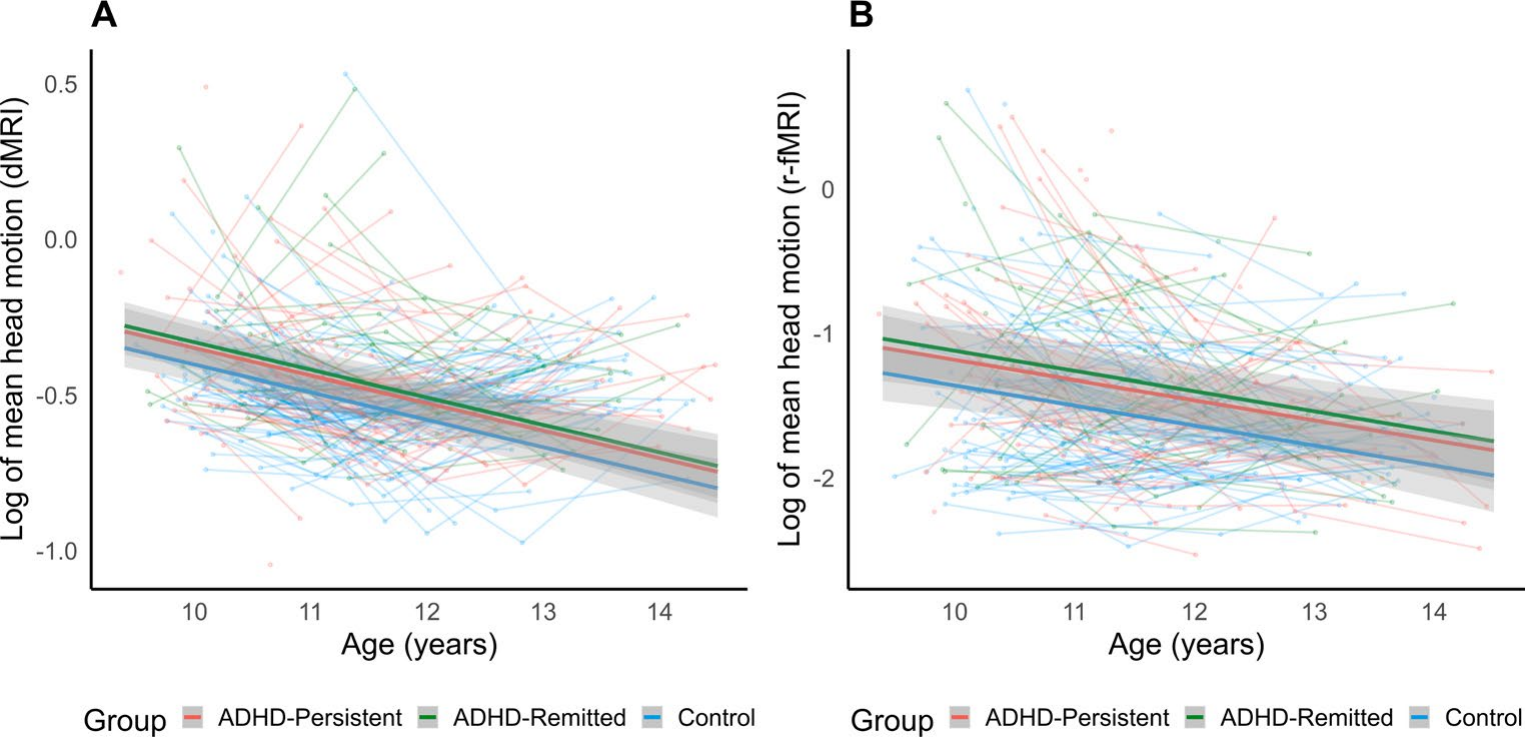
Effects of persistent and remitted ADHD status on longitudinal change in framewise displacement with age. *Note*. Figure depicts model trajectory of head motion (framewise displacement) change during (**A**) dMRI and (**B**) r-fMRI scans, plotted over raw motion values for each subject. Head motion decreases over age can be observed in ADHD-persistent (red), ADHD-remitted (green) and Control (blue) groups. Shaded areas represent 95% confidence intervals for best fitting model

To address Aim 2, further analysis investigated potential differences in trajectories of head motion over age between participants in remission from ADHD and those with persistent ADHD. There were no demographic differences between remitted and persistent ADHD groups except in ADHD medication use (*p* < .001, see Table S11), whereby those in the ADHD-persistent group had a higher rate of medication use than the ADHD-remitted and Control groups. After assessing fit statistics of nested GAMMs, Model 3 was chosen as the best-fitting model (see Table S12). In the final model, a significant age effect was observed for FD values across the sample, with head motion decreasing linearly with age for both dMRI and r-fMRI (*p* < .001; see Table 3; Fig. 2). For r-fMRI, a significant group main effect was observed between ADHD-persistent and Control groups, where participants with persistent ADHD displayed higher FD scores across the age range (*p* = .040). In contrast, no significant group difference was observed between persistent and remitted ADHD groups (*p* = .533). Post hoc tests indicated the ADHD-remitted group also displayed significantly higher FD than controls (estimate = 0.24, *SE* = 0.09, *p* = .011). For dMRI, there was similarly no observed differences between remitted and persistent ADHD groups (*p* = .584), and post hoc tests indicated that participants in remission from ADHD had higher FD than the Control group (estimate = 0.07, *SE* = 0.03, *p* = .020), however the difference between persistent-ADHD and Control groups was not significant (*p* = .066). Models explain 10% and 9% of the variance in mean FD during r-fMRI and dMRI, respectively. When further dividing the remitted ADHD group by whether remission occurred by wave 1 or wave 3, consistent results were found between persistent ADHD and control groups, and there were no differences between persistent ADHD and either ADHD remission group (see Table S13). Post hoc tests indicated the early ADHD-remitted group displayed significantly higher FD than controls during r-fMRI (*p* = .004) but not diffusion MRI (*p* = .630).

**Table 3:** Summary statistics of models demonstrating ADHD remission/persistent effects of head motion over age

MRI	Parametric coefficients	Estimate	*SE*	*t*-value	*p*-value
Diffusion	(Intercept)	-0.49	0.03	-15.63	< 0.001
	ADHD-Persistent vs. Control	-0.05	0.03	-1.84	0.066
	ADHD-Persistent vs. Remitted	0.02	0.03	0.55	0.584
	Scanner Upgrade	0.22	0.03	6.58	< 0.001
	Sex (Female vs. Male)	0.00	0.03	0.15	0.880
	ADHD Medication Use	0.04	0.04	1.00	0.317
	Smooth term	*edf*	*Ref.df*	*F*-value	*p*-value
	*s*(Age)	1	1	50.52	< 0.001
Resting state functional	Parametric coefficients	Estimate	*SE*	*t*-value	*p*-value
	(Intercept)	-1.41	0.09	-15.13	< 0.001
	ADHD-Persistent vs. Control	-0.17	0.08	-2.06	0.040
	ADHD-Persistent vs. Remitted	0.06	0.10	0.62	0.533
	Scanner Upgrade	0.05	0.09	0.53	0.595
	Sex (Female vs. Male)	0.08	0.08	1.04	0.301
	ADHD Medication Use	-0.17	0.11	-1.54	0.124
	Smooth term	*edf*	*Ref.df*	*F*-value	*p*-value
	*s*(Age)	1	1	16.43	< 0.001

*Note* Adjusted *R*^*2*^ 0.10 for diffusion MRI and 0.09 for resting state fMRI. Post hoc testing indicated that participants with ADHD in remission are statistically different to Control participants in head motion during both r-fMRI (Estimate = 0.24, *SE* = 0.09, *p* = .011) and diffusion MRI (Estimate = 0.07, *SE* = 0.03, *p* = .020)

*SE* = Standard Error, *edf* = estimated degrees of freedom, *Ref.df* = reference degrees of freedom

## Discussion

This study longitudinally investigated the trajectory of change in MRI-based head motion in children with and without ADHD. The relationship between ADHD remission and change in head motion was also explored. Numerous studies have investigated in-scanner motion in typical children throughout various stages of development cross-sectionally (Baum et al., 2018; Power et al., 2012; Satterthwaite et al., 2012). Researchers have also become increasingly interested in head motion in children with ADHD and focused on symptoms associated with in-scanner motion (Dziemian et al., 2022; Kong et al., 2014; Thomson et al., 2021). Recent evidence suggests that the greater motion in those with ADHD compared to controls could contribute to substantial inconsistencies in MRI research, introducing spurious bias and leading to data loss (Aoki et al., 2018; Pardoe et al., 2016). However, studies have yet to investigate intra-individual changes in head motion in either typically developing children or children with ADHD longitudinally. The current study found that while head motion in both typically developing children and children with ADHD decreased as children aged, children with ADHD had consistently greater motion. Children in remission from ADHD continued to display high head motion, despite presenting with reduced symptoms of ADHD possibly due to persistent differences in cognition.

Longitudinal analyses revealed that for both resting-state fMRI and dMRI, head motion in typically developing children significantly decreased in intensity and/or frequency as children aged. These findings align with previous cross-sectional research demonstrating that in-scanner motion steadily declines over development (Baum et al., 2018; Power et al., 2012; Satterthwaite et al., 2012; Wilke, 2012). Given the impact motion can have on fMRI and dMRI metrics, these findings highlight that a degree of caution should be exercised when making inferences on development, as some of the variance of interest may be accounted for by developmental changes in motion and the related artifact.

A similar decrease in head motion during MRI scanning was observed in children with ADHD, however motion was significantly greater in this group compared to controls across the studied age range. These findings are largely consistent with cross-sectional research (Pardoe et al., 2016; Thomson et al., 2021). Current literature suggests ADHD symptoms including hyperactivity, impulsivity and reduced sustained attention relate to in-scanner motion (Dziemian et al., 2022; Kong et al., 2014; Thomson et al., 2021), and decrease over development (Biederman et al., 2000; Willcutt, 2012), highlighting the possibility that children who have remitted from ADHD will exhibit lower levels of motion in the scanner. This hypothesis was tested in the current study. Interestingly, while both ADHD-in-remission and persistent-ADHD groups had significantly greater motion during r-fMRI than controls, no significant difference was observed between ADHD groups in either motion level or rate of decrease in head motion with age. These findings align with previous literature suggesting that while individuals may no longer meet criteria for an ADHD diagnosis, many continue to exhibit sub-threshold symptoms and difficulties in cognitive functioning associated with ADHD (Halperin et al., 2008; Thomson et al., 2020). Some of these cognitive domains (such as sustained attention) are linked to head motion (Thomson et al., 2021) and may explain the continued elevation in head motion in the ADHD in remission group. Similar results were obtained for dMRI, with the ADHD-in-remission group showing consistently higher head motion than controls across the age range, at a level comparable to individuals with persistent ADHD. Researchers should thus be aware that a motion bias (in terms of data quality and retention) may persist when studying brain structure and function in groups in remission from ADHD.

In a supplementary analysis separating the ADHD-in-remission group into early and later remitters, both remission groups showed comparable head motion to individuals with persistent ADHD. However, the early, but not later, remission group showed higher head motion than the control group across the age range. ADHD remission is complex; it is known that remission type, stability of remission status and age of remission can vary widely over development and into early adulthood (Biederman et al., 2000; Sibley et al., 2022). With diagnostic information in the current study only up to age 14, the long-term stability of persistent and remission status is unclear. In addition, diagnostic interview was conducted every 3 years. Future longitudinal work with high frequency diagnostic testing and following up in later adolescence or early adulthood is needed to determine the association between factors such as remission age and stability and head motion trajectories.

The current study had several strengths including the use of longitudinal motion and clinical data, allowing examination of intra-individual change in head motion over time with diagnosis, however has some limitations which raise new questions for future research. Pre-MRI training (i.e., mock scanner/simulator training) can significantly decrease motion during MRI collection (de Bie et al., 2010; Simhal et al., 2021). All participants completed practice MRI training prior to scans at every timepoint, however a limitation of the current study is that it could not account for the effect of increased familiarity with the scanning environment (over the longitudinal collection) on motion reductions. Supplementary analysis of a subset of 18 participants at their first and second scans, respectively (matched on age, sex and diagnostic group), show no differences in head motion due to number of scans attended (see Table S14), however comprehensive investigation of this question should be the focus of a future accelerated longitudinal study. Additionally, there is mixed evidence that other conditions such as anxiety are linked to in-scanner motion (Klaming et al., 2015) which was not considered in the current work. Future studies can longitudinally examine how within-individual fluctuations in anxiety might relate to MRI head motion. ADHD medication use was not associated with MRI head motion in main or supplementary analyses. However, the study design was not optimized to specifically test the effect of medication on motion, and conclusions about the effect of ADHD medication use on in-scanner motion require evidence from targeted studies such as randomized controlled trials. Finally, scanner upgrade was included as a covariate in all models and showed a significant association with FD during dMRI. This finding is consistent with previous work and may relate to longitudinal scanner drift (Thieleking et al., 2021). Further examination of potential effects of software upgrade and scanner drift on calculated movement parameters (as well as derived r-fMRI and dMRI measures more broadly) would be valuable for longitudinal investigations.

## Conclusion

In summary, current MRI research has revealed in-scanner head motion is more frequent and/or extreme in younger children, and that movement decreases as children age. Findings indicate that while motion decreases in both typically developing and ADHD groups as children age, those with ADHD display consistently greater head motion than controls during both resting-state fMRI and diffusion MRI sequences. The current study further found this trajectory of change in head motion is not altered when ADHD remission occurs, but rather children in remission from ADHD continue to display motion similar to that of children with persistent ADHD. Findings concur with evidence of persistent ADHD effects regardless of remission status and confirm the need for improved motion mitigation techniques to study true age effects and prevent ADHD-related biases.

## Electronic supplementary material

Below is the link to the electronic supplementary material.

Supplementary Material 1

## Data Availability

Full information and access for data from the Children’s Attention Project and Neuroimaging of the Children’s Attention Project cohorts are available via LifeCourse: https://lifecourse.melbournechildrens.com/cohorts/cap-and-nicap/. The study’s ethics approval conditions do not permit public archiving of anonymized study data, however readers seeking access to the data should contact the authors, the local ethics committee at the Royal Children’s Hospital (Melbourne, Australia) or follow the data access guide via LifeCourse (https://lifecourse.melbournechildrens.com/data-access/) and access will be granted in accordance with ethical procedures governing the reuse of clinical data, including completion of a formal data sharing agreement and approval of the local ethics committee.

## Code availability

Data were prepared for MCFLIRT according to documentation from the FMRIB Software Library (FSL, Version 6.0.0; Jenkinson et al., 2002). All code implemented for analysis were based on detailed R documentation for generalized additive mixed modelling from https://www.rdocumentation.org/packages/mgcv/versions/1.9-0/topics/gamm.
