# Supplementary material for: Changes in MRI head motion across development: typical development and ADHD
Source: Brain Imaging Behav. 2024 Aug 27;18(5):1144–52. doi: 10.1007/s11682-024-00910-w (PMC11582210; doi:10.1007/s11682-024-00910-w)
Supplement: Supplementary file 1 — Supplementary Material 1 [file 11682_2024_910_MOESM1_ESM.docx]

**Changes in MRI head motion across development: typical development and ADHD**

**Supplementary materials**

**Model parameters**

To assess effects of age and diagnostic status on motion during resting-state functional MRI (fMRI) and diffusion MRI (dMRI), the following models were tested in order of increasing complexity:

1. FD~1+ADHDMedicationUse+Sex+ScanUpgrade
2. FD~*s*(Age,k=4)+ADHDMedicationUse+Sex+ScanUpgrade
3. FD~*s*(Age,k=4)+Group+ADHDMedicationUse+Sex+ScanUpgrade
4. FD~*s*(Age,k=4)+Group+*s*(Age,k=4,by=Group,m=1)+ADHDMedicationUse+Sex+ScanUpgrade

To ensure the relationship between age and mean framewise displacement (FD) was not restricted to fitting a linear model, a penalized smoothing spline was used with a basis dimension (k) of 4, allowing for a maximum complexity of a cubic model (van Duijvenvoorde et al., 2019). In model 4, the addition of by=Group allows for different smooths for ADHD and Control groups, and m=1 penalizes these difference smooths (by group) based on the first derivative of the smooth to help with model identifiability.

**Supplementary analysis of ADHD symptom subdomains**

Supplementary analysis investigated the potential effect of symptom levels in each of the inattentive and hyperactivity/impulsivity subdomains on head motion during resting-state fMRI and dMRI. In this analysis, inattention and hyperactivity/impulsivity criteria of The Diagnostic Interview Schedule for Children at wave 1 (Shaffer et al., 2000) in participants with ADHD were used as variables of interest in generalized additive mixed models (in place of group in the formula from the Statistical Analysis section of the main methods). Results revealed no significant effect of number of inattention and hyperactivity/impulsivity symptoms on resting state fMRI or diffusion MRI FD within the ADHD group (see Tables S3 and S4).

**Analysis of aim 2 (effect of ADHD remission status on head motion) after exclusion of four participants with inconsistent ADHD diagnostic status**

Four participants received an ADHD diagnosis at recruitment and wave 3, but were subthreshold at wave 1. These individuals were included in the persistent ADHD group for the analysis for aim 2 due to their meeting of ADHD criteria at wave 3. For completeness, all analyses for aim 2 were rerun after exclusion of these four participants, with no changes to results (see Table S5).

**Analysis of aims 1 and 2 after notch filtering of head motion values for respiration effects**

Given recent work showing that respiration may inflate resting state functional MRI head motion estimates (Fair et al., 2020), models investigating the effect of age and diagnostic group on head motion during functional MRI were rerun after applying a notch filter to correct respiration induced artifacts. The filter was built and applied using the *iirnotch* and *filtfilt* functions of the SciPy python package (RRID:SCR_008058) with parameters of central cutoff frequency=0.31Hz and bandwidth =0.43Hz. As no respiratory belt was used during MRI scans, central cutoff frequency and the bandwidth parameters were used following Fair et al. (2020) who had the same mean age as the current study (11.6 years). After filtering framewise displacement values for resting state functional MRI scans, models were rerun, demonstrating consistent results to the main findings (see Tables S6–7).

**Supplementary analysis of ADHD medication use**

Analyses were rerun after the exclusion of 28 participants with ADHD taking ADHD medication. Results for aim 1 (effect of age and ADHD diagnosis on head motion) were consistent with primary analyses (see Table S8); head motion decreased as age increased, and children with ADHD displayed greater framewise displacement than controls over the age range. Results for aim 2 (effect of ADHD remission status on head motion) were also largely consistent with main findings; head motion remained elevated over the age range in children in remission from ADHD compared to participants in the control group (see Table S9). We note, however, that after exclusion of participants taking ADHD medication the persistent ADHD group showed significantly higher head motion across the age range for resting state functional MRI (*p*=.033) but not diffusion MRI (*p*=.072). This difference from main analyses is likely due to the fact that 24 of the 28 participants excluded on the basis of medication use were in the ADHD persistent group. The consistency of findings to main analyses after the exclusion of participants taking ADHD medication (apart from the aforementioned exception) is also consistent with independent samples t-tests showing that head motion is not significantly different between children with ADHD taking ADHD medication and not taking ADHD medication at any wave (see Table S10). A possible reason that no medication-related effects were observed in the current study is that in wave 1 participants with ADHD taking medication showed more severe symptoms on the Diagnostic Interview Schedule for Children (Shaffer et al., 2000) than participants with ADHD not taking medication for their symptoms (hyperactivity-impulsivity symptoms: *t*(84)=-3.95, p<.001; inattention symptoms: *t*(84)=-2.08, p=.041). This may have meant that despite potential positive effects of ADHD medication on reducing head motion, the negative effect of increased symptom levels in this group resulted in no overall difference in head motion between medication and non-medication ADHD groups.

**Analysis of aim 2 (effect of ADHD remission status on head motion) after separating ADHD in remission into early and later remission groups**

As a supplementary analysis, models for aim 2 were rerun after separating the ADHD in remission group into early remission (participants who met diagnostic criteria at recruitment but not at waves 1 and 3) and later remission (participants who met diagnostic criteria at recruitment and wave 1 but not wave 3) groups. Persistent ADHD, early ADHD remission and late ADHD remission groups did not differ in head motion during resting state functional or diffusion MRI over the age range (see Table S11), consistent with the results from the main analysis. Head motion during diffusion MRI did not differ across groups. A notable finding is that persistent ADHD and early ADHD remission groups showed higher FD during resting state functional MRI than the control group, while the late ADHD remission group did not.

**Table S1**

*ADHD medication use by collection wave*

| Medication, *n* (% medication group) | Wave 1 | Wave 2 | Wave 3 |
| --- | --- | --- | --- |
| Methylphenidate | 16 (73%) | 14 (70%) | 6 (86%) |
| Methylphenidate, Clonidine | 4 (18%) | 4 (20%) | 1 (14%) |
| Atomoxetine | 2 (9%) | 2 (10%) | 0 (0%) |
| Any ADHD Medication | 22 (100%) | 20 (100%) | 7 (100%) |

*Note.* Percentage indicates percentage of participants taking each medication out of the total number of participants on ADHD medication at each wave.

**Table S2**

*Fit indices (AIC, BIC and LRT) for Aim 1 models (ADHD and Control groups)*

| MRI | Model | *df* | AIC | BIC | logLik | Model comparison | Likelihood ratio | *p* |
| --- | --- | --- | --- | --- | --- | --- | --- | --- |
| Diffusion | 1 | 7 | -99 | -71 | 56.6 |  |  |  |
|  | 2 | 9 | -141 | -104 | 79.37 | 1 vs 2 | 45.54 | <.001 |
|  | **3** | 10 | -143 | -103 | 81.73 | 2 vs 3 | 4.73 | .030 |
|  | 4 | 12 | -139 | -91 | 81.73 | 3 vs 4 | 0.001 | .999 |
| Resting state functional | 1 | 7 | 743 | 771 | -364.57 |  |  |  |
|  | 2 | 9 | 733 | 769 | -357.49 | 1 vs 2 | 14.17 | <.001 |
|  | **3** | 10 | 723 | 763 | -351.45 | 2 vs 3 | 12.08 | <.001 |
|  | 4 | 12 | 727 | 775 | -351.38 | 3 vs 4 | 0.14 | .934 |

*Note*. Bold denotes selected model. *df* = degrees of freedom, AIC = Akaike information criterion, BIC = Bayesian information criterion, logLik = log likelihood.

**Table S3**

*Summary statistics of ADHD models for effects of inattention symptoms on head motion over age*

| MRI | Parametric coefficients | Estimate | *SE* | *t*-value | *p* |
| --- | --- | --- | --- | --- | --- |
| Diffusion | (Intercept) | -0.54 | 0.05 | -10.33 | <.001 |
|  | Inattention | 0.01 | 0.01 | 0.83 | .408 |
|  | Scanner Upgrade | 0.20 | 0.05 | 4.00 | <.001 |
|  | Sex (Female vs Male) | 0.03 | 0.04 | 0.78 | .436 |
|  | ADHD Medication Use | 0.03 | 0.04 | 0.66 | .513 |
|  | Smooth term | *edf* | *Ref.df* | *F*-value | *p*-value |
|  | *s*(Age) | 1 | 1 | 22.87 | <.001 |
| Resting state functional | Parametric coefficients | Estimate | *SE* | *t*-value | *p* |
|  | (Intercept) | -1.45 | 0.15 | -9.73 | <.001 |
|  | Inattention | 0.02 | 0.02 | 1.23 | .222 |
|  | Scanner Upgrade | 0.08 | 0.14 | 0.57 | .569 |
|  | Sex (Female vs Male) | 0.01 | 0.12 | 0.12 | .907 |
|  | ADHD Medication Use | -0.23 | 0.12 | -1.90 | .059 |
|  | Smooth term | *edf* | *Ref.df* | *F*-value | *p*-value |
|  | *s*(Age) | 1 | 1 | 12.61 | <.001 |

*Note.* Inattention symptom criteria scores based on The Diagnostic Interview Schedule for Children at wave 1. Models show no significant effect of initial inattention symptom severity on head motion during resting-state fMRI or diffusion MRI. Adjusted *R^2^* 0.06 for diffusion MRI and 0.10 for resting state fMRI. *SE* = Standard Error, *edf* = estimated degrees of freedom, *Ref.df* = reference degrees of freedom.

**Table S4**

*Summary statistics of ADHD models for effects of hyperactive/impulsive symptoms on head motion over age*

| MRI | Parametric coefficients | Estimate | *SE* | *t*-value | *p* |
| --- | --- | --- | --- | --- | --- |
| Diffusion | (Intercept) | -0.52 | 0.04 | -11.88 | <.001 |
|  | Hyperactivity/impulsivity | 0.01 | 0.01 | 0.75 | .453 |
|  | Scanner Upgrade | 0.20 | 0.05 | 4.00 | <.001 |
|  | Sex (Female vs Male) | 0.04 | 0.04 | 0.88 | .378 |
|  | ADHD Medication Use | 0.02 | 0.04 | 0.42 | .673 |
|  | Smooth term | *edf* | *Ref.df* | *F*-value | *p*-value |
|  | *s*(Age) | 1 | 1 | 23.17 | <.001 |
| Resting state functional | Parametric coefficients | Estimate | *SE* | *t*-value | *p* |
|  | (Intercept) | -1.41 | 0.13 | -10.85 | <.001 |
|  | Hyperactivity/impulsivity | 0.02 | 0.02 | 1.12 | .263 |
|  | Scanner Upgrade | 0.08 | 0.14 | 0.59 | .556 |
|  | Sex (Female vs Male) | 0.03 | 0.12 | 0.24 | .813 |
|  | ADHD Medication Use | -0.26 | 0.13 | -2.06 | .041 |
|  | Smooth term | *edf* | *Ref.df* | *F*-value | *p*-value |
|  | *s*(Age) | 1 | 1 | 13.04 | <.001 |

*Note.* Hyperactivity/impulsivity symptom criteria scores based on The Diagnostic Interview Schedule for Children at wave 1. Models show no significant effect of initial hyperactivity/impulsivity symptom severity on head motion during resting-state fMRI or diffusion MRI. Adjusted *R^2^* 0.06 for diffusion MRI and 0.09 for resting state fMRI. *SE* = Standard Error, *edf* = estimated degrees of freedom, *Ref.df* = reference degrees of freedom.

**Table S5**

*Summary statistics of models demonstrating ADHD remission/persistent effects of head motion over age after removal of participants with inconsistent ADHD diagnostic status*

| MRI | Parametric coefficients | Estimate | *SE* | *t*-value | *p* |
| --- | --- | --- | --- | --- | --- |
| Diffusion | (Intercept) | -0.48 | 0.03 | -14.22 | <.001 |
|  | ADHD-Persistent vs Control | -0.06 | 0.03 | -1.90 | .058 |
|  | ADHD-Persistent vs Remitted | 0.02 | 0.03 | 0.44 | .662 |
|  | Scanner Upgrade | 0.20 | 0.04 | 5.32 | <.001 |
|  | Sex (Female vs Male) | 0.00 | 0.03 | -0.12 | .904 |
|  | ADHD Medication Use | 0.03 | 0.04 | 0.86 | .389 |
|  | Smooth term | *edf* | *Ref.df* | *F*-value | *p* |
|  | *s*(Age) | 1 | 1 | 30.24 | <.001 |
| Resting state functional | Parametric coefficients | Estimate | *SE* | *t*-value | *p* |
|  | (Intercept) | -1.37 | 0.10 | -13.78 | <.001 |
|  | ADHD-Persistent vs Control | -0.22 | 0.09 | -2.39 | .017 |
|  | ADHD-Persistent vs Remitted | 0.03 | 0.11 | 0.25 | .805 |
|  | Scanner Upgrade | 0.05 | 0.09 | 0.54 | .588 |
|  | Sex (Female vs Male) | 0.08 | 0.08 | 1.04 | .299 |
|  | ADHD Medication Use | -0.20 | 0.11 | -1.80 | .072 |
|  | Smooth term | *edf* | *Ref.df* | *F*-value | *p* |
|  | *s*(Age) | 1 | 1 | 16.43 | <.001 |

*Note.* Replication of results from Aim 2 (Table 3) after excluding four participants who met diagnostic criteria at recruitment and wave 3 but were subthreshold at wave 1. Adjusted *R^2^* 0.11 for diffusion MRI and 0.10 for resting state fMRI. Post hoc testing indicated that participants with ADHD in remission are statistically different to Control participants in head motion during both resting state functional MRI (Estimate = 0.24, *SE* = 0.09, *p* = .010) and diffusion MRI (Estimate = 0.07, *SE* = 0.03, *p* = .021). *SE* = Standard Error, *edf* = estimated degrees of freedom, *Ref.df* = reference degrees of freedom.

**Table S6**

*Summary statistics of models demonstrating ADHD group effects on head motion during functional MRI over age after notch filtering*

| Resting state functional | Parametric coefficients | Estimate | *SE* | *t*-value | *p* |
| --- | --- | --- | --- | --- | --- |
|  | (Intercept) | -1.37 | 0.08 | -16.90 | <.001 |
|  | Group (ADHD vs Control) | -0.22 | 0.07 | -3.07 | .002 |
|  | Scanner Upgrade | 0.05 | 0.09 | 0.63 | .531 |
|  | Sex (Female vs Male) | 0.06 | 0.07 | 0.79 | .428 |
|  | ADHD Medication Use | -0.17 | 0.11 | -1.54 | .124 |
|  | Smooth term | *edf* | *Ref.df* | *F*-value | *p*-value |
|  | *s*(Age) | 1 | 1 | 16.35 | <.001 |

*Note.* Replication of results for resting state functional MRI data from Aim 1 (Table 2) after applying a notch filter for respiratory effects. Adjusted *R^2^* 0.09. *SE* = Standard Error, *edf* = estimated degrees of freedom, *Ref.df* = reference degrees of freedom.

**Table S7**

*Summary statistics of models demonstrating ADHD remission/persistent effects on head motion during functional MRI over age after notch filtering*

| Resting state functional | Parametric coefficients | Estimate | *SE* | *t*-value | *p* |
| --- | --- | --- | --- | --- | --- |
|  | (Intercept) | -1.40 | 0.09 | -14.94 | <.001 |
|  | ADHD-Persistent vs Control | -0.19 | 0.09 | -2.19 | .030 |
|  | ADHD-Persistent vs Remitted | 0.08 | 0.10 | 0.77 | .440 |
|  | Scanner Upgrade | 0.05 | 0.09 | 0.58 | .566 |
|  | Sex (Female vs Male) | 0.06 | 0.08 | 0.84 | .402 |
|  | ADHD Medication Use | -0.15 | 0.11 | -1.33 | .186 |
|  | Smooth term | *edf* | *Ref.df* | *F*-value | *p* |
|  | *s*(Age) | 1 | 1 | 15.84 | <.001 |

*Note.* Results for resting state functional MRI data from Aim 2 (Table 3) after applying a notch filter for respiratory effects. Adjusted *R^2^* 0.09. Post hoc testing indicated that participants with ADHD in remission are statistically different to Control participants in head motion during resting state functional MRI after notch filtering (Estimate = 0.26, *SE* = 0.09, *p* = .005). *SE* = Standard Error, *edf* = estimated degrees of freedom, *Ref.df* = reference degrees of freedom.

**Table S8**

*Summary statistics of models demonstrating ADHD group effects on head motion over age after removal of participants taking ADHD medication*

| MRI | Parametric coefficients | Estimate | *SE* | *t*-value | *p* |
| --- | --- | --- | --- | --- | --- |
| Diffusion | (Intercept) | -0.50 | 0.03 | -18.82 | <.001 |
|  | Group (ADHD vs Control) | -0.05 | 0.02 | -2.26 | .024 |
|  | Scanner Upgrade | 0.21 | 0.04 | 5.83 | <.001 |
|  | Sex (Female vs Male) | 0.02 | 0.03 | 0.64 | .526 |
|  | Smooth term | *edf* | *Ref.df* | *F*-value | *p* |
|  | *s*(Age) | 1 | 1 | 38.98 | <.001 |
| Resting state functional | Parametric coefficients | Estimate | *SE* | *t*-value | *p* |
|  | (Intercept) | -1.39 | 0.08 | -16.94 | <.001 |
|  | Group (ADHD vs Control) | -0.21 | 0.07 | -2.84 | .005 |
|  | Scanner Upgrade | 0.06 | 0.09 | 0.66 | .508 |
|  | Sex (Female vs Male) | 0.08 | 0.08 | 1.05 | .296 |
|  | Smooth term | *edf* | *Ref.df* | *F*-value | *p* |
|  | *s*(Age) | 1 | 1 | 14.78 | <.001 |

*Note.* Results from Aim 1 (Table 2) after excluding participants with ADHD taking ADHD medication at any wave. Adjusted *R^2^* 0.08 for diffusion MRI and 0.10 for resting state fMRI. *SE* = Standard Error, *edf* = estimated degrees of freedom, *Ref.df* = reference degrees of freedom.

**Table S9**

*Summary statistics of models demonstrating ADHD remission/persistent effects on head motion over age after removal of participants taking ADHD medication*

| MRI | Parametric coefficients | Estimate | *SE* | *t*-value | *p* |
| --- | --- | --- | --- | --- | --- |
| Diffusion | (Intercept) | -0.49 | 0.03 | -14.83 | <.001 |
|  | ADHD-Persistent vs Control | -0.05 | 0.03 | -1.81 | .072 |
|  | ADHD-Persistent vs Remitted | 0.02 | 0.04 | 0.66 | .513 |
|  | Scanner Upgrade | 0.21 | 0.04 | 4.96 | <.001 |
|  | Sex (Female vs Male) | 0.00 | 0.02 | -0.09 | .930 |
|  | Smooth term | *edf* | *Ref.df* | *F*-value | *p* |
|  | *s*(Age) | 1 | 1 | 28.62 | <.001 |
| Resting state functional | Parametric coefficients | Estimate | *SE* | *t*-value | *p* |
|  | (Intercept) | -1.39 | 0.10 | -13.61 | <.001 |
|  | ADHD-Persistent vs Control | -0.20 | 0.09 | -2.14 | .033 |
|  | ADHD-Persistent vs Remitted | 0.06 | 0.11 | 0.50 | .618 |
|  | Scanner Upgrade | 0.07 | 0.09 | 0.82 | .415 |
|  | Sex (Female vs Male) | 0.07 | 0.08 | 0.92 | .356 |
|  | Smooth term | *edf* | *Ref.df* | *F*-value | *p* |
|  | *s*(Age) | 1 | 1 | 14.81 | <.001 |

*Note.* Results from Aim 2 (Table 3) after excluding participants with ADHD taking ADHD medication at any wave. Adjusted *R^2^* 0.10 for diffusion MRI and 0.10 for resting state fMRI. Post hoc testing indicated that participants with ADHD in remission are statistically different to Control participants in head motion during both resting state functional MRI (Estimate = 0.26, *SE* = 0.10, *p* = .009) and diffusion MRI (Estimate = 0.08, *SE* = 0.03, *p* = .013). *SE* = Standard Error, *edf* = estimated degrees of freedom, *Ref.df* = reference degrees of freedom.

**Table S10**

*Head motion between participants in the ADHD group taking and not taking ADHD medication*

|  |  | FD by ADHD group | |  |
| --- | --- | --- | --- | --- |
| MRI | Wave | Taking ADHD medication | Not taking ADHD medication | Statistics |
| Diffusion | 1 | -0.29 (0.28) | -0.38 (0.20) | *t*(84)=-1.68, *p*=.097 |
|  | 2 | -0.51 (0.22) | -0.49 (0.29) | *t*(84)=0.31, *p*=.756 |
|  | 3 | -0.39 (0.13) | -0.44 (0.14) | *t*(50)=-0.91, *p*=.366 |
| Resting state functional | 1 | -1.40 (0.77) | -1.03 (0.79) | *t*(75)=1.82, *p*=.073 |
|  | 2 | -1.5 (0.48) | -1.38 (0.65) | *t*(82)=0.75, *p*=.457 |
|  | 3 | -1.87 (0.35) | -1.55 (0.51) | *t*(49)=1.61, *p*=.113 |

*Note.* Independent samples t tests indicate no significant differences in framewise displacement (FD) between participants in the ADHD group taking and not taking ADHD medication at any wave. FD log transformed due to high degree of rightward skew.

**Table S11**

*Demographic characteristics of participants for Aim 2 (ADHD-persistent, ADHD-remitted and Control groups)*

|  |  | ADHD | | |  | |  | |  |
| --- | --- | --- | --- | --- | --- | --- | --- | --- | --- |
| MRI | Measure | Remitted | Persistent | Control | | Test statistic | | *p* | |
| Diffusion | Participants | 35 | 63 | 83 | | - | | - | |
|  | Males, *n* (%) | 23 (66%) | 52 (83%) | 49 (59%) | | 9.33 | | .009^a^ | |
|  | Age, *M (SD)* |  |  |  | |  | |  | |
|  | Wave 1 | 10.4 (0.4) | 10.4 (0.5) | 10.4 (0.4) | | 0.90 | | .914 | |
|  | Wave 2 | 11.7 (0.5) | 11.7 (0.6) | 11.7 (0.5) | | 0.05 | | .956 | |
|  | Wave 3 | 13.2 (0.5) | 13.3 (0.7) | 13.2 (0.5) | | 0.81 | | .447 | |
|  | IQ, *M* (*SD*) | 94.3 (14.8) | 95.3 (12.8) | 103.6 (13.7) | | 9.20 | | <.001^b^ | |
|  | SES, *M* (*SD*) | 1027 (36) | 1015 (43) | 1019 (47) | | 0.85 | | .429 | |
|  | ADHD Medication use, *n* (%) | 4 (11%) | 24 (38%) | 0 (0%) | | 18.18 | | <.001^c^ | |
| Resting state functional | Participants | 33 | 61 | 82 | | - | | - | |
|  | Males, *n* (%) | 22 (67%) | 51 (84%) | 48 (59%) | | 10.32 | | .006^a^ | |
|  | Age, *M (SD)* |  |  |  | |  | |  | |
|  | Wave 1 | 10.4 (0.4) | 10.4 (0.5) | 10.4 (0.4) | | 1.19 | | .830 | |
|  | Wave 2 | 11.7 (0.5) | 11.7 (0.6) | 11.7 (0.5) | | 0.04 | | .957 | |
|  | Wave 3 | 13.2 (0.5) | 13.3 (0.7) | 13.2 (0.5) | | 0.81 | | .447 | |
|  | IQ, *M* (*SD*) | 95.1 (14.8) | 95.4 (13.0) | 103.8 (13.6) | | 8.52 | | <.001^b^ | |
|  | SES, *M* (*SD*) | 1026 (36) | 1015 (43) | 1018 (47) | | 0.71 | | .494 | |
|  | ADHD Medication use, *n* (%) | 4 (12%) | 24 (39%) | 0 (0%) | | 40.91 | | <.001^c^ | |

*Note*. *M* = mean*, SD* = standard deviation, IQ = intelligence quotient, SES = socioeconomic status. a: ADHD-persistent > Control, ADHD-persistent = ADHD-remitted, ADHD-remitted = Control; b: Control > ADHD-persistent = ADHD-remitted; c: ADHD-persistent > ADHD-remitted > Control.

**Table S12**

*Fit indices (AIC, BIC and LRT) for Aim 2 models (ADHD-persistent, ADHD-remitted and Control groups)*

| MRI | Model | *df* | AIC | BIC | logLik | Model comparison | Likelihood ratio | *p* |
| --- | --- | --- | --- | --- | --- | --- | --- | --- |
| Diffusion | 1 | 7 | -98 | -70 | 55.93 |  |  |  |
|  | 2 | 9 | -138 | -102 | 78.23 | 1 vs 2 | 44.60 | <.001 |
|  | **3** | 11 | -142 | -97 | 81.82 | 2 vs 3 | 7.17 | .028 |
|  | 4 | 14 | -136 | -80 | 82.12 | 3 vs 4 | 0.61 | .895 |
| Resting state functional | 1 | 7 | 720 | 748 | -352.98 |  |  |  |
|  | 2 | 9 | 710 | 746 | -346.13 | 1 vs 2 | 13.70 | .001 |
|  | **3** | 11 | 703 | 747 | -340.34 | 2 vs 3 | 11.58 | .003 |
|  | 4 | 14 | 708 | 764 | -340.22 | 3 vs 4 | 0.25 | .970 |

*Note*. Bold denotes selected model. *df* = degrees of freedom, AIC = Akaike information criterion, BIC = Bayesian information criterion, logLik = log likelihood.

**Table S13**

*Summary statistics of models demonstrating ADHD remission/persistent effects of head motion over age, separated by early and later remission status*

| MRI | Parametric coefficients | Estimate | *SE* | *t*-value | *p* |
| --- | --- | --- | --- | --- | --- |
| Diffusion | (Intercept) | -0.49 | 0.03 | -15.41 | <.001 |
|  | ADHD-Persistent vs Control | -0.05 | 0.03 | -1.86 | .064 |
|  | ADHD-Persistent vs Early Remission | 0.00 | 0.04 | 0.10 | .921 |
|  | ADHD-Persistent vs Later Remission | 0.03 | 0.05 | 0.66 | .507 |
|  | Scanner Upgrade | 0.22 | 0.03 | 6.53 | <.001 |
|  | Sex (Female vs Male) | 0.00 | 0.03 | 0.06 | .949 |
|  | ADHD Medication Use | 0.04 | 0.04 | 0.99 | .321 |
|  | Smooth term | *edf* | *Ref.df* | *F*-value | *p* |
|  | *s*(Age) | 1 | 1 | 49.65 | <.001 |
| Resting state functional | Parametric coefficients | Estimate | *SE* | *t*-value | *p* |
|  | (Intercept) | -1.43 | 0.09 | -15.43 | <.001 |
|  | ADHD-Persistent vs Control | -0.17 | 0.08 | -2.00 | .046 |
|  | ADHD-Persistent vs Early Remission | 0.18 | 0.13 | 1.38 | .168 |
|  | ADHD-Persistent vs Later Remission | 0.10 | 0.14 | 0.71 | .481 |
|  | Scanner Upgrade | 0.05 | 0.09 | 0.55 | .585 |
|  | Sex (Female vs Male) | 0.10 | 0.08 | 1.36 | .174 |
|  | ADHD Medication Use | -0.18 | 0.11 | -1.62 | .106 |
|  | Smooth term | *edf* | *Ref.df* | *F*-value | *p* |
|  | *s*(Age) | 1 | 1 | 16.69 | <.001 |

*Note.* Results from Aim 2 (Table 3) after separating ADHD in remission group into early remission (participants who met diagnostic criteria at recruitment but not at waves 1 and 3) and late remission (participants who met diagnostic criteria at recruitment and wave 1 but not wave 3) groups. Adjusted *R^2^* 0.10 for diffusion MRI and 0.10 for resting state fMRI. Post hoc testing indicated that participants with ADHD in remission by wave 1 (mean age 10.4 years) are statistically different to Control participants in head motion during resting state functional MRI (Estimate = 0.34, *SE* = 0.12, *p* = .004) but not diffusion MRI (Estimate = 0.06, *SE* = 0.04, *p* = .155). Participants with ADHD who remitted from their symptoms between waves 1 and 3 are not statistically different to Control participants in head motion during resting state functional MRI (Estimate = 0.27, *SE* = 0.14, *p* = .054) or diffusion MRI (Estimate = 0.09, *SE* = 0.05, *p* = .070). Early and later ADHD in remission groups did not differ in head motion during resting state functional MRI (Estimate = 0.08, *SE* = 0.17, *p* = .651) or diffusion MRI (Estimate = 0.03, *SE* = 0.06, *p* = .630). *SE* = Standard Error, *edf* = estimated degrees of freedom, *Ref.df* = reference degrees of freedom.

**Table S14**

*Independent samples t-test of mean FD between age-matched participants who have attended one vs two scans*

|  | Framewise displacement, *M (SD)* | |  |  |  |
| --- | --- | --- | --- | --- | --- |
| MRI | Wave 1 group | Wave 2 group | t-value | *df* | *p*-value |
| Diffusion | 0.67 (0.15) | 0.69 (0.34) | 0.23 | 34 | .820 |
| Resting state functional | 0.40 (0.44) | 0.34 (0.27) | -0.57 | 34 | .574 |

*Note*. Head motion comparison conducted between 18 participants attending their first scan at wave 1 and 18 participants attending their second scan at wave 2 (matched on age, sex and diagnostic group), suggest no significant differences in head motion due to number of scans attended. Comparison conducted between waves 1 and 2 only given the potential effects of scanner upgrade between waves 2 and 3 on FD during diffusion MRI. *M* = mean, SD = standard deviation.
